# Analysis of Yersinia pseudotuberculosis Isolates Recovered from Deceased Mammals of a German Zoo Animal Collection

**DOI:** 10.1128/JCM.03125-20

**Published:** 2021-05-19

**Authors:** J. A. Hammerl, N. vom Ort, A. Barac, C. Jäckel, L. Grund, S. Dreyer, C. Heydel, A. Kuczka, H. Peters, S. Hertwig

**Affiliations:** a German Federal Institute for Risk Assessment, Department of Biological Safety, Berlin, Germany; b Der Grüne Zoo Wuppertal, Wuppertal, Germany; c Justus-Liebig University Giessen, Institute of Hygiene and Infectious Diseases of Animals, Giessen, Germany; d Chemisches- und Veterinäruntersuchungsamt Rhein-Ruhr-Wupper, Fachgebiete Pathologie und Bakteriologie/Mykologie, Krefeld, Germany; University of Tennessee at Knoxville

**Keywords:** *Yersinia*, genome, diversity, virulence factor, antimicrobial resistance, plasmid, prophage, zoo animals

## Abstract

Yersinia pseudotuberculosis is an important pathogen for both humans and animals. It can infect livestock, as well as pets and wild animals. During recent years, a number of reports have described the isolation of Y. pseudotuberculosis from zoo animals, mainly birds and mammals, for which the infection was mostly lethal. Between 2005 and 2019, there were at least 17 cases of deceased mammals, belonging to five different species, which suffered from a Y. pseudotuberculosis infection at the Zoo Wuppertal, Germany. Since only scarce information exists on the properties of Y. pseudotuberculosis from zoo animals, we characterized eight isolates, covering all infected species, in detail. All isolates were members of biotype 1, but belonged to five serotypes, five sequence types (STs), and seven core-genome multilocus sequence types (cgMLSTs). Using pulsed-field gel electrophoresis (PFGE) analysis and whole-genome sequencing (WGS), the seven isolates could be discriminated from each other. They differed significantly regarding their virulence genes and mobile genetic elements. While the virulence plasmid pYV existed in all serotypes (five isolates), a complete high-pathogenicity island (HPI) was detected only in the serotypes O:1a, O:1b, and O:13 (four isolates), but not in O:2a and O:2b. Similarly, the content of other plasmids and prophages varied greatly between the isolates. The data demonstrate that the deceased mammals were infected by seven individual isolates and not by a single type predominating in the zoo animals.

## INTRODUCTION

Yersinia pseudotuberculosis and Yersinia enterocolitica cause the enteric disease yersiniosis, the third most common bacterial enteritis in Europe (1, 2). Together with Yersinia pestis, the causative agent of plague, Y. pseudotuberculosis and Y. enterocolitica represent the human-pathogenic species of the genus, whereas 16 other *Yersinia* species are considered to be nonpathogenic for humans (3). Interestingly, at the DNA level, Y. pseudotuberculosis is much more closely related to Y. pestis than to Y. enterocolitica, although yersiniosis and plague are very different diseases with respect to the infection route and clinical picture. Indeed, Y. pestis is a recently emerged clone of Y. pseudotuberculosis that diverged within the last 1,500 to 20,000 years (4). Thus, it may not seem surprising that, despite that all pathogenic *Yersinia* strains share some chromosomal virulence genes (e.g., *ail* for the attachment invasion locus) and a similar 70-kb virulence plasmid (pYV), Y. pseudotuberculosis possesses a high-pathogenicity island (HPI) encoding the siderophore yersiniabactin, which also exists in Y. pestis, but only in bio-/serotype 1B/O:8 strains in in Y. enterocolitica (5). Another difference from Y. enterocolitica concerns the natural reservoirs of these species. The presence of Y. enterocolitica is clearly associated with pigs and most yersiniosis infections are caused by the consumption of pork contaminated with this species (6). In contrast, the host spectrum of Y. pseudotuberculosis is more diverse, as this species is not only a human pathogen but can also infect a broad spectrum of animals, e.g., livestock, pets, wild animals, and zoo animals (7, 8). In fact, the number of reports describing the infection of zoo animals with Y. pseudotuberculosis is constantly increasing. Yersinia pseudotuberculosis is often implicated in lethal epidemics in zoo animals (9). Besides birds, which seem to be quite often infected by Y. pseudotuberculosis (10–13), some mammals have also been affected, e.g., breeding monkeys, meerkats, and a paca (14–16).

Regrettably, most isolates in the aforementioned studies were not characterized in detail. In this work, eight Y. pseudotuberculosis isolates recovered from deceased mammals in the zoo in Wuppertal, Germany were analyzed and compared by bio-/serotyping, multi locus sequence typing (MLST), PFGE, and WGS. The study shows that the Y. pseudotuberculosis isolates belong to several types and that they differ significantly regarding their virulence gene content and mobile genetic elements.

## MATERIALS AND METHODS

### Isolation and cultivation of Y. pseudotuberculosis from zoo animals.

Isolation of Y. pseudotuberculosis YE00106 and YE00066 was conducted at the IHIT (Institute of Hygiene and Infectious Diseases of Animals, Justus-Liebig University Giessen) by streaking out samples onto blood agar plates (Merck) supplemented with 5% sheep blood, water-blue metachrome-yellow lactose agar (according to GASSNER; Sifin), brilliant-green phenol-red lactose sucrose agar (Merck), and *Yersinia*-selective agar (Merck) with *Yersinia*-selective supplement (Oxoid, Dassel, Germany) followed by an incubation at 37°C for 24 and 48 h. (We chose two incubation times because *Y. pseudotuberculosis* is a rather slow growing pathogen which, however, can be easily overgrown by other bacteria.) Anaerobic bacteria were further cultivated on Zeissler (Merck) and Schaedler (Becton and Dickinson) agar supplemented with 5% sheep blood for 72 h at 37°C under anaerobic conditions using a jar and Anaerogen sachets (Oxoid).

The isolation of 19-YE00057, 19-YE00065, and 19-YE00070 was performed at the CVUA-RRW (Chemisches- und Veterinäruntersuchungsamt Rhein-Ruhr-Wupper) by streaking out samples onto blood agar plates supplemented with 5% sheep blood (Merck), MacConkey agar 3 (MC3), brilliant-green phenol-red lactose sucrose agar followed by an incubation at 37°C for 24 and 48 h.

For initial species identification, *Yersinia*-like colonies were subjected to matrix-assisted laser desorption ionization–time of flight mass spectrometry (MALDI-TOF MS) analysis using a Microflex LT/SH (Bruker Daltonics, Bremen Germany) with the direct transfer protocol. For further analysis, all isolates were transferred to the Consultant Laboratory for *Yersinia* at the German Federal Institute for Risk Assessment (BfR), Berlin, Germany. If not otherwise indicated, the Y. pseudotuberculosis isolates were cultivated in lysogeny broth (LB) at 28°C under shaking conditions (200 to 225 rpm) (17).

### Determination of the species, biotype, and serotype.

To confirm the species Y. pseudotuberculosis, the isolates were subjected to the following biochemical tests: Voges Proskauer, indol, citrate, sorbitol, sucrose, rhamnose, esculin, and melibiose. For the determination of the biotype, the metabolism of melibiose and raffinose, as well as the metabolic conversion of citrate, were studied (18). Serotyping of the isolates was conducted by multiplex PCR targeting different regions of the O-antigen cluster of Y. pseudotuberculosis (19). Representative isolates of the BfR strain collection were used as controls.

### Antimicrobial susceptibility testing.

Antimicrobial susceptibility testing was performed by broth microdilution following the CLSI guidelines (CLSI M07-A10). For this investigation, the standardized EUVSEC and EUVSEC2 plate format (Trek Diagnostic Systems Inc., the Netherlands) was used. As some isolates were recovered from host animals treated with amoxicillin and ceftiofur, a possible resistance against amoxicillin (0.03 to 4.0 mg/liter) and ceftiofur (0.008 to 0.5 mg/liter) was examined using individually composed plates (Trek Diagnostic Systems Inc.). As a quality control, the Escherichia coli strain ATCC 25922 was used. MICs were interpreted as previously described (20).

### Pulsed-field gel electrophoresis.

Pulsed-field gel electrophoresis (PFGE) was conducted for macrorestriction analysis of the isolates and the determination of the plasmid content, as previously described (21). Macrorestriction analysis was performed by incubating agarose plugs of the isolates with XbaI (Roche Diagnostics, Mannheim, Germany) for 4 h at 37°C. Electrophoresis was carried out with an electric field of 6 V/cm and an angle of 120°. Pulsed-field agarose gels (0.8%) were run at 14°C in 0.5× Tris-borate-EDTA (TBE) buffer. Pulse times ranged from 4 to 40 s for 21 h. For the determination of the plasmid content, the agarose plugs were incubated with S1 nuclease for 45 min at 37°C. Here, the pulse times ranged from 1 to 25 s for 17 h. In general, the *Salmonella* Braenderup strain H9812 was used as a standard marker (21).

### Analysis of the low-calcium response of the *Yersinia* virulence plasmid pYV.

The low-calcium response of pYV was determined by growing the isolates on Congo red magnesium-oxalate agar (CR-MOX test), as previously described (22, 23). Isolates showing a growth inhibition at 37°C were rated as positive.

### Whole-genome sequencing, genome annotation, and *in silico* typing.

Whole-genome sequencing (WGS) of Y. pseudotuberculosis isolates was conducted with genomic DNA (gDNA) extracted with the Purelink Genomic DNA minikit (Invitrogen, Germany) from liquid cultures inoculated from a single colony grown on Columbia agar supplemented with 5% sheep blood (bioMérieux, Germany) as recommended by the manufacturer. The purity and quality parameters of the gDNA necessary for WGS were determined with the Nanodrop 1000 Spectrophotometer V3.8 (VWR, Germany) and the Qubit 2.0 fluorometer (Thermo Fisher Scientific, Germany) according to the standard protocol. gDNA samples meeting the Illumina Inc. specifications were further used for DNA sequencing library preparation with the Nextera XT DNA sample preparation kit (Illumina, CA, USA) according to the manufacturer’s protocol. Short-read, paired-end sequencing was performed in 2 × 251 cycles on the Illumina MiSeq benchtop using the Illumina MiSeq Reagent v3 600-cycle kit (24). After demultiplexing and trimming of the raw reads using the “BakCharak” pipeline (https://gitlab.com/bfr_bioinformatics/bakcharak) of the German Federal Institute for Risk Assessment (BfR), *de novo* assemblies using the full SPAdes algorithm of the PATRIC database (www.patricbrc.org) were performed (25). Final annotation of the bacterial genomes was conducted using the automated submission portal (https://submit.ncbi.nlm.nih.gov/) of the National Center for Biotechnology Information (NCBI).

In general, individual bioinformatic tools (i.e., ResFinder v 3.0 [26], PlasmidFinder v 2.0 [27], and MLST v 2.0 [28]) of the Center for Genomic Epidemiology (https://cge.cbs.dtu.dk/services/) were used for *in silico* typing of the Y. pseudotuberculosis genomes. The phylogenetic relationship of the isolates was determined using CSI phylogeny v 1.4 (https://cge.cbs.dtu.dk/services/CSIPhylogeny/) and visualized with the FigTree software (release 1.4.3) (29). Prophage prediction was carried out using PHASTER (https://phaster.ca/) (30). Further in-depth analyses were carried out using CLC Genomics Workbench 9.5.2 (Qiagen, Germany) and DS-Gene v2.5 (Accelrys Inc., USA).

### Data availability.

The genomes of the Y. pseudotuberculosis isolates were deposited in GenBank under the accession numbers 18-YE00011 (WGGE00000000), 18-YE00012 (WGGF00000000), 19-YE00056 (WGGH00000000), 19-YE00057 (WGGI00000000), 19-YE00065 (WGGK00000000), 19-YE00066 (WGGL00000000), 19-YE00070 (WGGP00000000), and 19-YE00106 (WMBG00000000).

## RESULTS

### Various mammals kept in separate enclosures in the zoo were infected.

Between 2005 and 2019, eight Y. pseudotuberculosis isolates were obtained from diseased animals (two black-headed spider monkeys [Ateles fusciceps], two Patagonian maras [Dolichotis patagonum], one Chacoan mara [Dolichotis salinicola], one pudu [Pudu puda; two strains] and one short-eared elephant shrew [Macroscelides proboscideus]) kept in separate locations at the Zoo Wuppertal (Fig. S1 in the supplemental material, Table 1). The map shows that the enclosures, for example those of the Chacoan maras and pudus, from which Y. pseudotuberculosis was isolated in 2019 are remote from each other. The animals showed different clinical symptoms (e.g., apathy, regenerative anemia, watery diarrhea). They all died, even though some of them were treated with amoxicillin or ceftiofur (Table 2). Testing of a number of antibiotics, including β-lactams (i.e., amoxicillin and ampicillin), however, did not give any indication of resistance to these antimicrobial agents, whereas six isolates showed a non-wild-type phenotype (resistance) against colistin (Table S1). Pathological examination of the animals revealed, in most cases, damage of internal organs that indicated microbial infection. Samples taken from the dead animals were investigated in terms of potential pathogens. From all animals, Y. pseudotuberculosis was isolated and characterized in detail. Two isolates (19-YE00057 and 19-YE00070) were recovered from the same pudu.

**TABLE 1:** Origin of the Y. pseudotuberculosis isolates analyzed in this study

Isolate	Source^*a*^	Host	Country	Year
18-YE00011	Spleen	Black-headed spider monkey	Germany	2018
18-YE00012	Spleen	Black-headed spider monkey	Germany	2018
19-YE00056	Brain	Chacoan mara	Germany	2019
19-YE00057	Liquor	Pudu	Germany	2019
19-YE00065	n.s.	Patagonian mara	Germany	2015
19-YE00066	Abscess	Patagonian mara	Germany	2016
19-YE00070	Liquor	Pudu	Germany	2019
19-YE00106	n.s.^2^	Short-eared elephant shrew	Germany	2005

a n.s., not specified; n.s.^2^, liver, spleen, kidney, lung, bowel, and brain were affected.

**TABLE 2:** Clinical symptoms, pathological findings, and therapeutic treatment of the animals

Isolate (host animal)	Clinical symptoms	Pathology	Treatment/medical procedures
18-YE00011 (Black-headed Spider monkey)	Apathy, death under anesthesia	High-grade multifocal necrotizing inflammation of liver and spleen
18-YE00012 (Black-headed Spider monkey)	Apathy, reduced appetite	High-grade hyperplasia of spleen and mesenteric lymph nodes	Antibiotic treatment (amoxcillin, ceftiofur); NSAID (meloxicam); Supportive treatment during anesthesia (fluid supplementation and vitamins)
19-YE00056 (Chacoan mara)	Found dead without previous clinical signs	Purulent-necrotizing hepatitis; splenitis and typhlocolitis
19-YE00057, 19-YE00070 (Pudu)	Watery diarrhea	Enterocolitis	Antibiotic treatment (amoxicillin); antiparasitic treatment (ivermectin), supportive treatment (fluid supplementation and oral electrolytes)
19-YE00065 (Patagonian mara)	Apathy, ataxia, paraparesis	Chronic subcutaneous phlegmona above the lumbar spine; necrotizing hepatitis and splenitis	Euthanasia
19-YE00066 (Patagonian mara)	Found dead without previous clinical signs	Multiple internal abscesses
19-YE00106 (Short-eared elephant shrew)	Apathy, death	Necrotizing hepatitis

### Eight isolates represent several Y. pseudotuberculosis groups.

**FIG 1:**
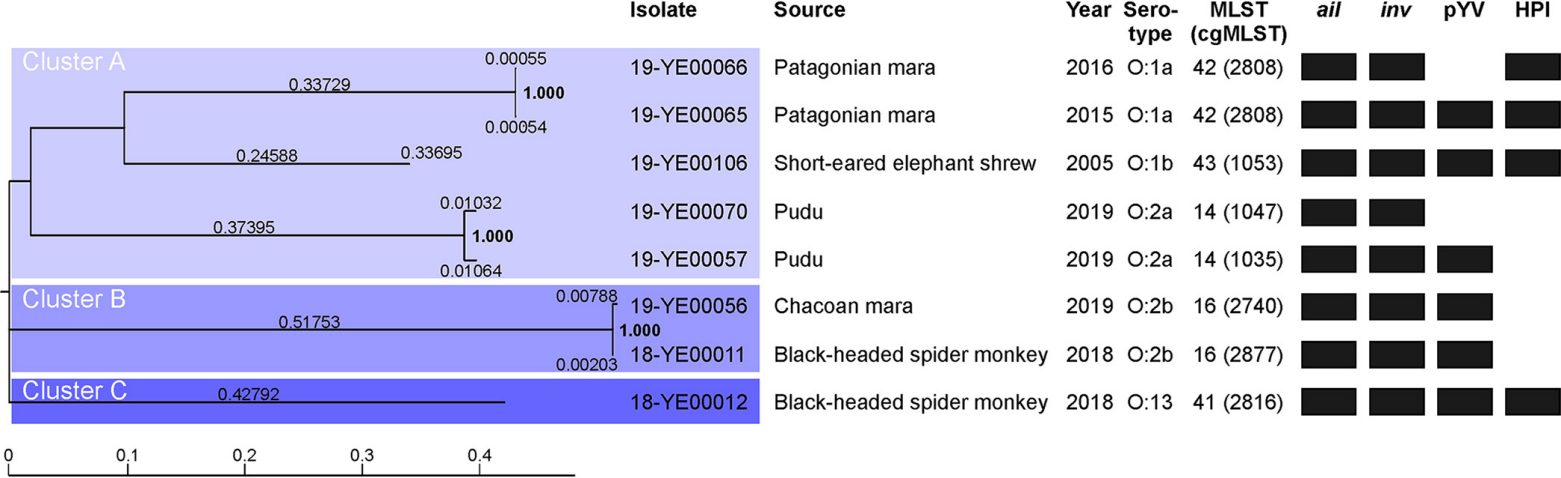
Phylogenetic relationship of the Y. pseudotuberculosis isolates. The phylogenetic relationship was calculated with CSI Phylogeny v1.4 using the following parameters: minimum depth at SNP positions = 10×; min. relative depth at SNP positions = 10×; minimum distance between SNPs (prune) = 10 bp; minimum SNP quality = 30; minimum read mapping quality = 25; and a minimal Z-score of 1.96. Heterozygous SNPs were ignored. The presence of virulence-associated DNA regions is indicated by black boxes.

We first determined the biotypes of the isolates. All of them belonged to biotype 1. The serotype was determined by PCR. The study showed that the eight isolates are members of five different serotypes, O:1a, O:1b, O:2a, O:2b, and O:13 (Fig. S2). Moreover, they have five different STs (MLST) and even seven core-genome multilocus sequent types (cgMLST) (Table 3). Except for 19-YE00065 and 19-YE00066, obtained from two Patagonian maras in 2015 and 2016, respectively, that both belong to serotype O:1a, ST 42 and cgMLST 2808, all other isolates could be distinguished from each other. This result was corroborated by PFGE macrorestriction analysis, which demonstrated distinct restriction patterns of most isolates (Fig. S3). They were also sequenced. Compared to the reference genome (18-YE00011: 6,220,334 bp), the sequencing data covered 4,188,287 bp (67.33%) that were found in all analyzed genomes. Phylogenetic analysis revealed the isolates belonged to three clearly different clusters (Fig. 1). Clustering was in good agreement with MLST typing except for 18-YE00012, whose MLST type (20) differs only in one allele from the MLST types 14 and 42, but belongs to a single cluster.

**TABLE 3:** Typing and virulence genes of the isolates

Isolate	18-YE00011	18-YE00012	19-YE00056	19-YE00057	19-YE00065	19-YE00066	19-YE00070	19-YE00106
Biotype	1	1	1	1	1	1	1	1
Serotype	O:2b	O:13	O:2b	O:2a	O:1a	O:1a	O:2a	O:1b
MLST type	16	41	16	14	42	42	14	43
cgMLST type	2,877	2,816	2,740	1,035	2,808	2,808	1,047	1,053
Virulence gene sequence identity^*a*^^,^^*b*^								
Chromosomal								
* ail*	99.63	99.63	99.63	99.63	100	100	99.63	99.63
* inv*	99.66	99.96	99.66	99.85	99.96	99.96	99.85	100
* ypmA*	ND	(+)	ND	(+)	(+)	(+)	(+)	(+)
* ypmB*	(+)	ND	(+)	ND	(+)	(+)	ND	(+)
* ypmC*	(+)	ND	(+)	ND	ND	ND	ND	ND
pYV								
* yadA*	(+)	(+)	(+)	(+)	(+)	ND	ND	(+)
* virF*	99.88	99.75	99.88	99.88	99.88	ND	ND	99.63
* yopE*	99.55	100	99.55	100	99.85	ND	ND	100
* yopH*	100	100	100	100	99.86	ND	ND	100
* yopT*	100	99.79	100	100	100	ND	ND	100
* yopM*	92.96	(+)	(+)	(+)	(+)	ND	ND	(+)
* yopO*	99.86	99.91	99.86	99.82	99.86	ND	ND	99.95
* yopP/J*	100	99.88	100	100	100	ND	ND	100
HPI								
* psn*	ND	100	ND	ND	100	100	ND	99.99
* irp1*	ND	99.97	ND	ND	99.99	99.99	ND	100
* irp2*	ND	99.97	ND	ND	100	100	ND	99.98
* ybtP*	ND	99.89	ND	ND	99.94	99.94	ND	99.94
* ybtQ*	ND	100	ND	ND	100	100	ND	100
* ybtA*	ND	100	ND	ND	100	100	ND	100
* ybtE*	ND	99.94	ND	ND	100	100	ND	100
* ybtS*	ND	100	ND	ND	100	100	ND	100

a For sequences that were completely detectable, percentage of the sequence identity to the reference is given.

b ND, not detected; (+), partial sequences identified.

### Virulence gene content.

**FIG 2:**
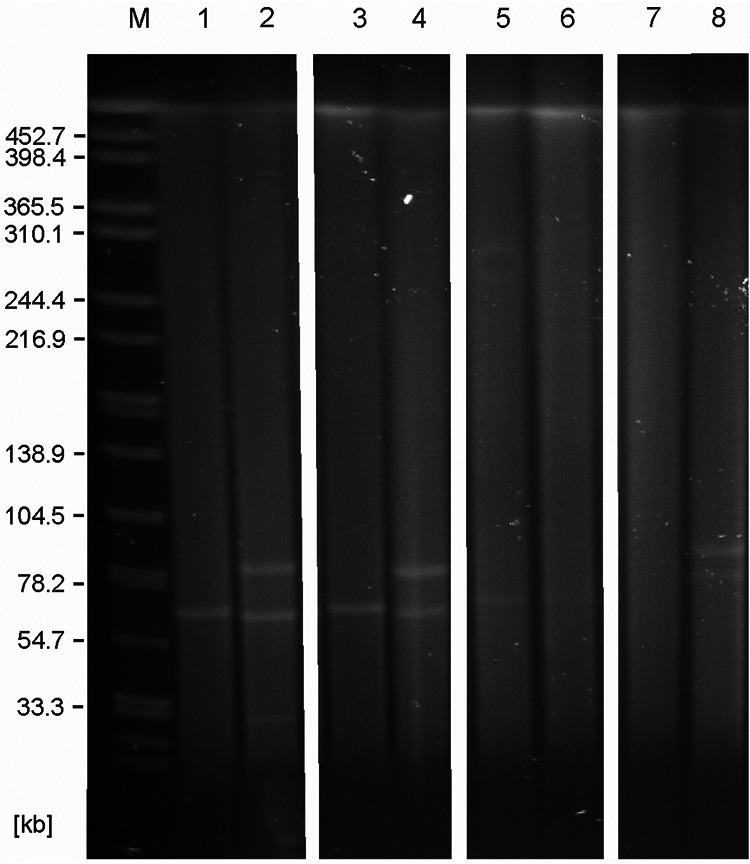
S1-nuclease PFGE profiles of the Y. pseudotuberculosis isolates. Lane M, molecular weight standard (*Salmonella* Braenderup H9812); lane 1, 18-YE00011; lane 2, 18-YE00012; lane 3, 19-YE00056; lane 4, 19-YE00057; lane 5, 19-YE00065; lane 6, 19-YE00066; lane 7, 19-YE00070, and lane 8, 19-YE00106.

The virulence of Y. pseudotuberculosis is caused by both chromosomal genes and the virulence plasmid pYV (Table 3). A plasmid of approximately 70 kb was detected in five isolates (18-YE00011, 18-YE00012, 19-YE00056, 19-YE00057, and 19-YE00065), whereas 19-YE00106 contained a plasmid that was several kilobases larger (Fig. 2). However, WGS confirmed the presence of genes located on pYV in all these isolates, e.g., *yadA*, encoding a collagen-binding protein important for autoagglutination, adherence to epithelial cells, and serum resistance, and *virF*, a transcriptional activator of the *Yersinia* virulence regulon (Table 3). In addition, a number of genes for *Yersinia* effector proteins (YopE, YopH, YopT, YopM, YopO, and YopP/J) were identified, which have a toxic effect and are secreted into eukaryotic cells by a type III secretion system (T3SS), thereby protecting the bacteria against the host’s immune system and enabling the proliferation and spread of the pathogen. In two isolates (19-YE00066 and 19-YE00070), pYV was not detected by S1-nuclease PFGE or WGS (Fig. 2, Table 3, Table 4). Cultivation of the pYV-positive isolates on Congo red-magnesium oxalate agar (CR-MOX) at 37°C revealed tiny red colonies, suggesting that the calcium response region of pYV was functional (data not shown). In contrast, the growth of the remaining isolates on CR-MOX at 37°C confirmed that they did not contain pYV.

**TABLE 4:** Mobile genetic elements (plasmids and prophages) of the isolates

Isolate	18-YE00011	18-YE00012	19-YE00056	19-YE00057	19-YE00065	19-YE00066	19-YE00070	19-YE00106
No. of plasmids	1	3	1	2	1	0	0	2
*inc*-group^*a*^	IncFII(Y)	IncFII(Y)	IncFII(Y)	IncFII(Y)	IncFII(Y)	ND	ND	IncFII(Y)
*inc*-group^*a*^	ND	ncFII	ND	ncFII	ND	ND	ND	ncFII
*inc*-group^*a*^	ND	[IncFII]	ND	ND	ND	ND	ND	ND
No. of prophages (no. of intact prophages)	5 (2)	7 (3)	4 (1)	8 (2)	5 (2)	5 (2)	2 (1)	4 (3)
Identity of intact prophages	RE_2010, *Salmonella* (NC_019488); SuMu, *Haemophilus* (NC_019455)	vB_SosS_Oslo, *Salmonella*, (NC_018279); SEN1, *Salmonella* (NC_029003); D108, *Escherichia* (NC_013594)	ENT90, *Erwinia* (NC_019932)	phiES15, *Cronobacter* (NC_018454); Fels_2, *Enterobacteria* (NC_010463)	phiES15, *Cronobacter* (NC_018454); RE_2010, *Salmonella* (NC_019488)	phiES15, *Cronobacter* (NC_018454); RE_2010, *Salmonella* (NC_019488)	RE_2010, *Salmonella* (NC_019488)	RE_2010, *Salmonella* (NC_019488); ENT90, *Erwinia* (NC_019932); vB_MhM_3927A2, *Mannheimia* (NC_028766)

a ND, not detected. Inc replicon types with sequence identities below 100% to the reference sequence are indicated by square brackets.

The chromosome of most enteropathogenic yersiniae harbors the genes *ail* (attachment invasion locus) and *inv* (invasin), which promote adherence to and invasion into eukaryotic cells. These important virulence genes were detected in all isolates. In contrast, a superantigenic toxin (YPM) existing in three variants (*ypmA*, *ypmB*, and *ypmC*) was not present in any of them. Some Y. pseudotuberculosis strains possess a high-pathogenicity island (HPI) encoding proteins for the biosynthesis, regulation, and transport of the siderophore yersiniabactin. Five genes of the HPI are mainly involved in the yersiniabactin system (*psn*, *irp1*, *irp2*, *ybtP*, and *ybtQ*). Psn is the outer membrane receptor for the siderophore, the genes *irp1* and *irp2* code for high-molecular-weight proteins involved in the nonribosomal synthesis of yersiniabactin, while ABC transporter proteins are encoded by *ybtP* and *ybtQ.* Four (18-YE00012, 19-YE00065, 19-YE00066, and 19-YE00106) of the eight isolates contained all five genes and additionally other genes (*ybtA*, *ybtE*, and *ybtS*) also important for yersiniabactin production. In the remaining four isolates, no yersiniabactin gene was detected. There are also sequences in the high-pathogenicity island that are responsible for genetic mobility. The gene *int* encodes an integrase that is similar to the integrase of phage P4, whereas the insertion element IS100 is involved in genomic rearrangements. Both genetic elements were found solely in the four isolates containing the yersiniabactin system.

In conclusion, the analysis of the virulence gene content revealed a high degree of heterogeneity in the eight isolates. Only three isolates (18-YE00012, 19-YE00065, and 19-YE00106) contained both the virulence plasmid pYV and important genes (*ail*, *inv*, complete HPI) located on the chromosome. In one (19-YE00066) and three (18-YE00011, 19-YE00056, and 19-YE00057) isolates, pYV and the yersiniabactin system, respectively, were missing, while 19-YE00070 lacked both. Considering the possibility that 19-YE00066 may have lost pYV during cultivation, the serotypes O:1 and O:13 possess more virulence genes (HPI and pYV) than serotype O:2 isolates, which are lacking HPI.

### Isolates carry numerous mobile genetic elements.

**FIG 3:**
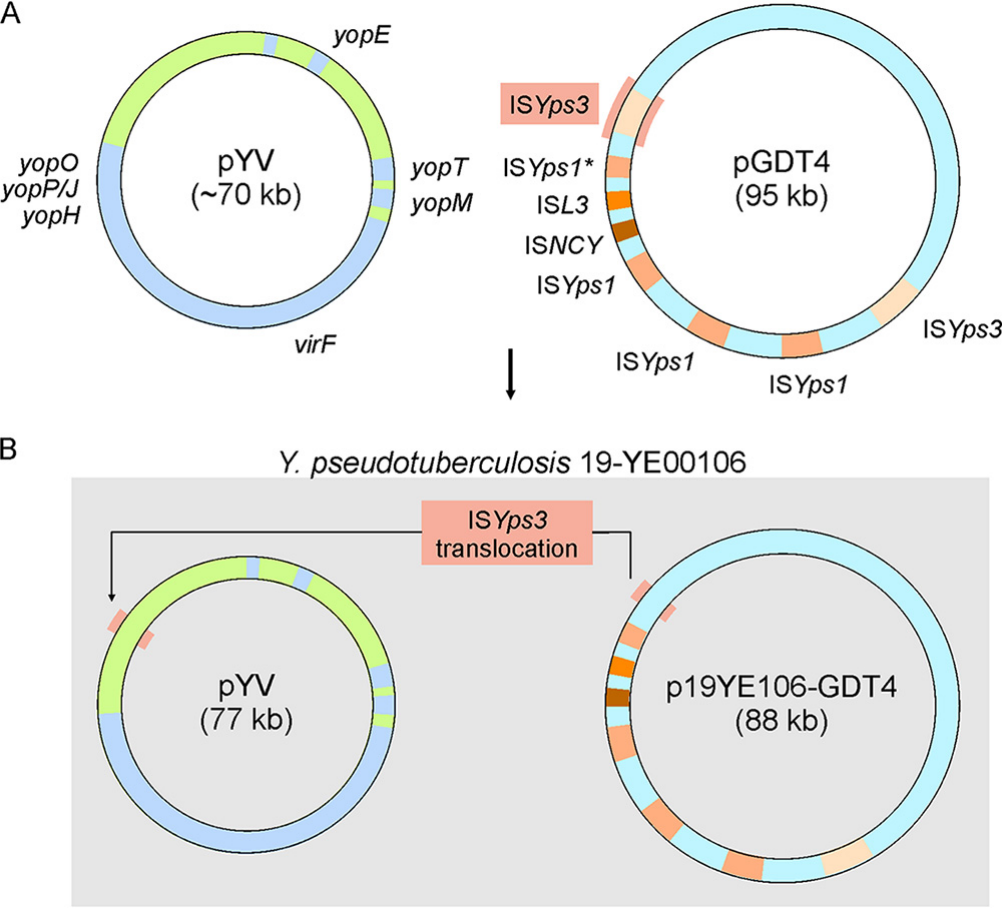
Translocation of one IS*Yps3* transposon of pGDT4 into pYV in the isolate 19-YE00106. (A) Schematic illustration of the plasmids pYV and pGDT4. (B) Both plasmids after translocation of the IS*Yps3* transposon.

S1-nuclease PFGE analysis revealed various numbers of plasmids in the isolates. Besides the 70-kb virulence plasmid, some of them contained one or two additional plasmids, approximately 30 kb and 85 kb in size (Fig. 2). Sequencing of plasmid preparations (see the Materials and Methods) showed that the three large plasmids of 18-YE00012, 19-YE00057, and 19-YE00106 are almost identical. They are very similar to the 95-kb conjugative plasmid pGDT4, which is able to mobilize parts of the Y. pseudotuberculosis chromosome and nonconjugative plasmids at 4°C (31). The plasmid of the zoo isolates revealed only a 7.3-kb deletion (nucleotide position 65,636 to 72,935 in pGDT4), encompassing one of the two copies of the IS*Yps3* transposon of pGDT4 (Fig. 3). This transposon harbors genes for a resolvase, SpnT protein, and a *recA* regulator RecX, which are important for recombination. The deletion may indicate a former transposition event. This assumption was confirmed by the finding, that the 7.3-kb fragment containing IS*Yps3* was detected in the virulence plasmid pYV of 19-YE00106, which therefore is significantly larger (77.1 kb) than the other virulence plasmids (Fig. 3B). It is notable that the fragment of pGDT4 was inserted in an IS3 transposase gene of pYV (Fig. 3).

The small plasmid (32.5 kb) of 18-YE00012 showed some similarities to the conjugative plasmid pLT (60 kb) of Yersinia ruckeri SC09 (CP025802.1), even though transfer genes are missing in the plasmid of the zoo isolate. However, it is conceivable that this plasmid can be mobilized by the pGDT4-related plasmid of 18-YE00012.

By WGS, up to nine prophage-related sequences were detected in each isolate, some of which were classified as intact (Table 4). Of these sequences, the most frequent prophage was similar to the temperate *Salmonella* myovirus RE-2010 (34.1 kb, NC_019488), which has the potential to laterally transfer genes between different *Salmonella* serovars via lysogenic conversion (32). The second most common prophage was related to the siphovirus phiES15 (39.9 kb, NC_018454) isolated from Cronobacter sakazakii (33). Other prophages classified as intact were similar to the temperate Erwinia amylovora myovirus ENT90 (29.5 kb, NC_019932), to the *Enterobacteria* phage Fels-2 (33.7 kb, NC_010463), which is closely related to *Salmonella* phage RE-2010 (NC_019488), or to E. coli phage D108 (37.2 kb, NC_013594) and Haemophilus parasuis phage SuMu (37.2 kb, NC_019455). The latter two myoviruses are particularly interesting because of their relationship to phage MU. Phage D108, indeed, is a transposable Mu-like phage that has been shown to generate apparently random chromosomal mutations (34). In addition, D108 is able to transfer DNA by generalized transduction (35).

## DISCUSSION

This study demonstrates that different Y. pseudotuberculosis types infected and probably killed various animals at Zoo Wuppertal. Though it cannot be excluded that other pathogens were also involved, it is very likely that the isolates played an important role in the origin of the observed diseases. The importance of Y. pseudotuberculosis for the infection of mammals, including nonhuman primates, has already been reported by other authors (14–16, 36, 37). However, to the best of our knowledge, this is the first study describing isolates recovered from mammals of unrelated taxa (New World monkeys, rodents, deer, and macroscelids) kept in the same zoo in such detail. Particularly useful for the characterization of the isolates were PFGE and WGS analysis, by which 19-YE00057 and 19-YE00070 could be clearly discriminated, despite that they exhibited the same bio/serotype and ST, and had been isolated from the same pudu in 2019. On the other hand, the only case in which two animals (Patagonian maras) may have been infected by the same isolate in 2015 and 2016 pertains to 19-YE00065 and 19-YE00066, which are very similar, revealing an identical ST and PFGE pattern. They only differ by the presence of the virulence plasmid pYV, which, however, is known to be rather unstable (38, 39). So it is quite possible that even though these two isolates were recovered in successive years, a transmission from one mara to the other may have occurred. Similarly, in 2005 an isolate had been obtained from a second deceased short-eared elephant shrew which was identical to 19-YE00106 apart from a missing virulence plasmid (data not shown). All other isolates were distinct and there was no indication of transmission between animals, which is not surprising in view of the spatial separation of the respective species. Moreover, the animals were infected by isolates belonging to different clonal lineages, the origins of which, however, are still unknown. This also applies to the two spider monkeys, both of which died in 2018. They were infected by 18-YE00011 and 18-YE00012, which are clearly different (Table 3).

Analysis of the isolates showed they all belong to biotype 1 but to several serotypes (O:1a [*n* = 2], O:1b [*n* = 1], O:2a [*n* = 2], O:2b [*n* = 2], and 13 [*n* = 1]). The serotypes O:1a and O:1b are the most common in Europe, Australasia, and North America (40). Moreover, serotype O:1 has been reported to cause infections of wild boars, pigs, and humans (41–43). Similarly, serotype O:2a was found to be associated with septicemia in hares (44). On the basis of its virulence gene content (pYV, HPI, and YPM), Y. pseudotuberculosis has been divided into six genetic groups (45). According to this classification, 18-YE00012, 19-YE00065, and 19-YE00106 belong to group 2 (HPI^+^, YPM^−^, pYV^+^), which is predominantly found in Europe as serotypes 1a and 1b, but may also include serotype 13, to which 18-YE00012 belongs. Isolates of genetic type 2 are highly pathogenic for humans (44). Three other isolates (18-YE00011, 19-YE00056, and 19-YE00057) are members of group 6 (HPI^−^, YPM^−^, pYV^+^), whereas 19-YE00066 (HPI^+^, YPM^−^, pYV^−^) and 19-YE00070 (HPI^−^, YPM^−^, pYV^−^) do not fit into this scheme. However, as mentioned above, these isolates might have lost their virulence plasmid and may therefore also belong to group 2 (19-YE00066) and group 6 (19-YE00070). In this case, the serotype of the isolates would perfectly fit to the group affiliation, since European serotype O:1 normally contains a complete HPI, while the island is generally missing in serotype O:2 (45). The YPM superantigenic toxin is exclusively present in the groups 1, 3, 4, and 5, but is mainly found in strains in the Far East (46), where none of the affected animals was ever kept. Thus, it is likely that the mammals in the Zoo Wuppertal were infected by local isolates. Moreover, the isolates, even though some of them lacked both HPI and YPM, probably had the potential to kill these animals. There was, however, no discernible correlation between the observed symptoms and the serotypes and their content of virulence genes.

It has already been reported that the virulence plasmid pYV, the HPI, and even large fragments of the bacterial chromosome can be horizontally transferred from one Y. pseudotuberculosis strain to another by the help of conjugative plasmids (17, 47). However, a pGDT4-related plasmid has only been described in one Yersinia similis strain (48). Thus, it is notable that three zoo isolates possessed such a plasmid, suggesting that it is more widespread than thought. Similarly, only scarce information exists on temperate Y. pseudotuberculosis phages that may also play a major role in horizontal gene transfer and recombination. In this study, numerous prophage sequences were identified in the isolates, some of which occurred in several of them. Most intriguingly, three prophages classified as intact are related to a phage that is able to transfer genes by lysogenic conversion, or else to Mu-like phages, which can cause genetic transposition. Although such activities have to be examined by further experiments, the numbers of conjugative plasmids and prophage sequences in the isolates suggest extensive modular shuffling within and gene exchange between them. This presumption is supported by the finding that members of each serotype contain a different composition of mobile genetic elements.

What can be done to prevent lethal Y. pseudotuberculosis infections of zoo animals in the future? One main problem is that individuals of nondomesticated species often mask clinical signs as long as possible in order to avoid appearing vulnerable (49). Therefore, clinical signs often do not become perceivable until the animal’s disease has progressed severely, making the therapy of the disease all the more challenging. Moreover, the necessity of general anesthesia for most diagnostic approaches in these zoological patients poses additional risks for weakened individuals and often prolongs the time needed to come to the specific diagnosis in the first place. The described cases in the pudu, the Chacoan mara, and the black-headed spider monkeys were the first observed in these species at Zoo Wuppertal. For this reason, it took some time for the diagnosis to become evident. Fecal samples do not always reveal shedding of *Yersinia* species. However, initial therapy of the pudu and the black-headed spider monkey with broad-spectrum antibiotics had been started long before Y. pseudotuberculosis was identified as the causative agent. Although ceftiofur administered to the black-headed spider monkey is a third-generation-cephalosporin, which is used for agents resistant to other beta-lactam antibiotics, it was not effective in this particular case. This is plausible, since it has been shown that *in vivo* efficacy of beta-lactam antibiotics can be insufficient, even if *in vitro* susceptibility of Y. pseudotuberculosis is high (50). In hindsight, fluoroquinolones would have been the antibiotic agents of choice, rather than beta-lactam antibiotics (50). The source of infection could not be determined in the above-mentioned cases, but rodent pest control measures have been increased at Zoo Wuppertal, and stable vaccines were fabricated and are administered yearly to the species that have been affected.

In conclusion, the management of Y. pseudotuberculosis infections in a zoo setting is difficult and the therapy of clinically diseased zoo animals is often elusive. Nevertheless, whenever this pathogen is suspected, fluoroquinolones should be used as the drug of choice to begin with, and antibiotic therapy should be adjusted according to microbial sensitivity as soon as possible (51).
